# Preparation and Evaluation of the Remineralization Effect of a Dental Varnish Containing Casein Phosphopeptide-Amorphous Calcium Phosphate with Fluoride (Cpp-Acpf) on White Spot Lesions of Permanent Teeth: An In Vitro Study

**DOI:** 10.34172/joddd.44108

**Published:** 2026-06-30

**Authors:** Sarah Uriat, Sina Gholami, Somayeh Khoramian Tusi, Faranak Salmannejad

**Affiliations:** ^1^Department of Pharmaceutics, Faculty of Pharmacy, Alborz University of Medical Sciences, Karaj, Iran; ^2^Department of Pediatric Dentistry, Faculty of Dentistry, Alborz University of Medical Sciences, Karaj, Iran

**Keywords:** Casein phosphopeptide-amorphous calcium phosphate, CPP-ACP, Dental caries, Dental materials, Fluorides, Tooth remineralization

## Abstract

**Introduction:**

White spot lesions (WSLs) are early indicators of enamel demineralization and require effective r e m i n e r a l i z a t i o n strategies to prevent progression. This in vitro study aimed to prepare and evaluate a dental varnish containing casein phosphopeptide-amorphous calcium phosphate with fluoride (CPP-ACPF) and to compare its remineralization efficacy with a commercial MI GC varnish and a control varnish.

**Methods:**

The dental varnish formulation was prepared. To evaluate its physicochemical properties and compare them with the MI varnish, density and viscosity measurements were conducted. To evaluate the effect of the varnishes on remineralization, 36 enamel specimens were obtained from the buccal surface of extracted human non-carious premolars. The specimens were randomly divided into three groups (n=12). Microhardness measurements of the tooth surface were taken at three stages: (1) baseline (sound enamel) before demineralization, (2) immediately after demineralization, and (3) after the remineralization period. Scanning electron microscopy (SEM) was used to examine the enamel surface morphology at each stage.

**Results:**

Enamel surface microhardness (Vickers hardness number, VHN) significantly decreased after demineralization (baseline: 310±12; after demineralization: 185±10; *P*<0.001). Following treatment, the prepared CPP-ACPF group exhibited the highest recovery (275±11 VHN), compared with MI varnish (245±13 VHN) and control (210±9 VHN). Repeated-measures mixed ANOVA confirmed significant differences between the groups (*P*<0.001), with the Tukey test showing that prepared CPP-ACPF varnish>MI varnish>control. Scanning electron microscopy revealed smoother and more uniform remineralized surfaces in the prepared CPP-ACPF group.

**Conclusion:**

The results indicated that the prepared CPP-ACPF varnish provided superior remineralization and could serve as a stable, cost-effective option for managing early enamel lesions.

## Graphical Abstract

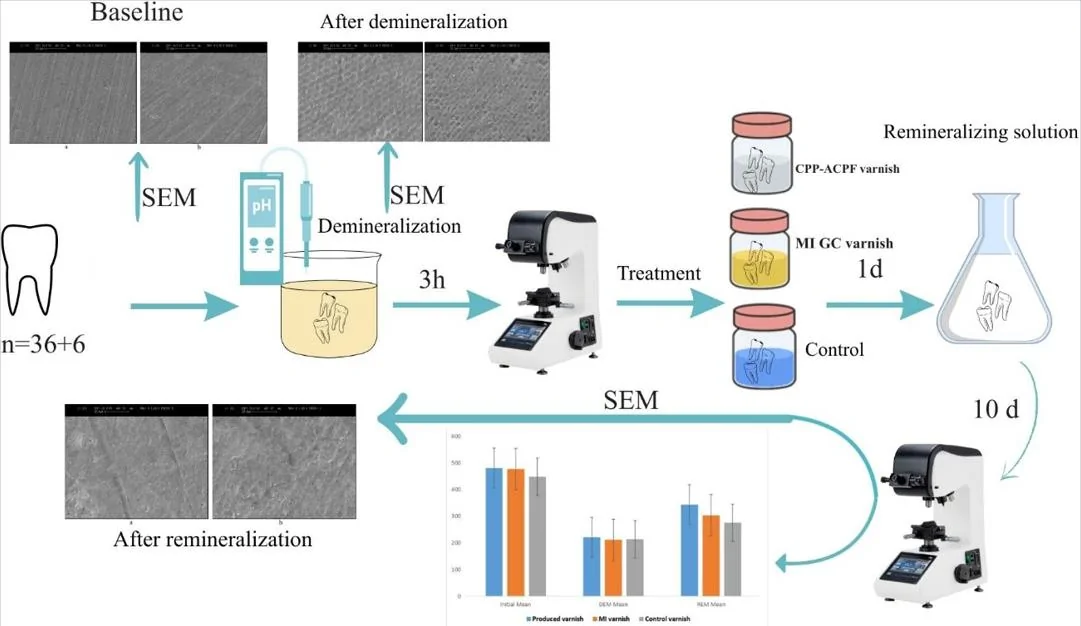

## Introduction

 White spot lesions (WSLs) are among the earliest and most common clinical manifestations of macroscopic enamel demineralization and are often considered precursors to cavitated carious lesions. These lesions appear as opaque, matte, whitish areas on the tooth surface and result from a disruption in the dynamic balance between two processes in enamel: demineralization (loss of minerals) and remineralization (re-uptake of minerals).^1^

 When the rate and extent of demineralization exceed the rate and extent of remineralization over a prolonged period, these structural alterations in enamel become clinically detectable. Demineralization primarily occurs when the oral pH falls below the critical threshold of 5.5; in such acidic conditions, generated by bacterial metabolism within dental plaque, hydrogen ions react with hydroxyapatite crystals, leading to the release of hydroxyl and phosphate ions.^2^

 These changes mostly occur in the subsurface layer of enamel, while the surface layer remains relatively intact during the initial stages. The accumulation of microscopic porosities in the subsurface alters the optical properties of enamel, giving the lesion its characteristic whitish, opaque appearance. If left untreated, the subsurface destruction progresses, and eventually, the collapse of the surface layer leads to the development of cavitated carious lesions.^3^

 Remineralization is a dynamic process in dental hard tissues, during which lost mineral ions—particularly calcium, phosphate, and hydroxyl—are re-incorporated into the crystalline lattice of enamel. The effectiveness of this process depends mainly on two factors: first, the saturation or supersaturation of saliva and dental plaque with calcium and phosphate ions; and second, the pH fluctuations of the oral environment, which shift between acidic and alkaline cycles throughout the day.^4^

 Saliva plays a crucial role in maintaining this balance through its bicarbonate-buffering system, protective proteins, and mineral content. Any reduction in salivary flow—for instance, in xerostomia or as a side effect of certain medications—can shift the balance toward demineralization.^5^

 In addition, frequent intake of fermentable sugars, poor oral hygiene, plaque accumulation in retentive sites such as around orthodontic brackets, and repeated consumption of acidic beverages shift the balance toward demineralization.^6^

 Fluoride is one of the key agents that facilitates remineralization. By replacing hydroxyl ions in the hydroxyapatite structure, fluoride forms fluorapatite, which exhibits lower solubility and greater acid resistance. Furthermore, calcium fluoride-like deposits formed on the tooth surface act as a reservoir of fluoride, releasing fluoride ions under acidic conditions. Fluoride also reduces acid production by inhibiting bacterial enzymatic activity in cariogenic biofilms.^7^

 In recent years, dental varnishes have gained increasing attention as one of the most effective approaches for the prevention and management of early enamel lesions. Varnishes adhere to teeth, allowing gradual release of active agents and prolonged contact with enamel. The 5% sodium fluoride varnish is commonly used to strengthen enamel by forming a calcium fluoride-like surface layer.^8^

 Clinically, the application of high-concentration fluoride may result in excessive remineralization of the lesion surface, thereby restricting ion penetration into the deeper layers. This highlights the need for adjunctive strategies that, in combination with fluoride, can effectively deliver sustained calcium and phosphate ions into the lesion’s depth. Bioactive alternatives such as CPP-ACPF provide a stable source of calcium, phosphate, and fluoride, enhancing deeper remineralization.^9-11^

 CPP-ACP, derived from milk protein, stabilizes calcium and phosphate ions near the enamel surface. It releases minerals in response to pH drops and provides buffering to protect enamel. Combined with fluoride (CPP-ACPF), it delivers fluoride to subsurface layers, enhancing remineralization.^12^

 Evidence indicates that CPP-ACPF is effective not only in the management of white spot lesions but also in improving discolored lesions ranging from yellow to brown. This property, combined with its lower risk of fluorosis compared with high-concentration fluoride, makes CPP-ACPF an attractive option for both preventive and therapeutic interventions. In this context, the development and local production of such varnishes could reduce costs, improve accessibility, and represent a significant step toward enhancing oral health at the population level.^13,14^

 Many previous studies have assessed CPP-ACPF’s remineralization potential compared to other treatments, highlighting its clinical relevance. The addition of CPP-ACP to the fluoride varnish significantly enhanced both penetration ability and protective effect on enamel.^15,16^

 Furthermore, an experimental study employing advanced imaging and elemental analysis techniques (micro-CT, FE-SEM, and EDX) evaluated the efficacy of two remineralizing agents, CPP-ACPF (MI varnish) and the self-assembling peptide P11-4 (Curodont FP), in the prevention and treatment of white spot lesions. In this study, the intervention groups exhibited significant improvements in lesion depth and mineral density.^17^

 This study aimed to compare CPP-ACPF and MI varnishes on extracted premolars, assessing enamel microhardness and microstructural morphology using a repeated-measures mixed ANOVA and post hoc Tukey tests.

## Methods

###  Materials

 CPP-ACP was procured from Shaanxi Dideu Medichem Company (Shaanxi, China). Polyvinyl acetate, hydrogenated rosin gum, xylitol, and Parafilm M^®^ were procured from Sigma-Aldrich Chemical Company (Missouri, USA). Ethanol, fumed silica, and sodium fluoride were obtained from Merck Millipore Company (Germany). Deionized water (Millipore Company, USA) was used for all solution preparations. The toffee flavor was gifted by the Toska Company (Tehran, Iran).

###  Preparation of Varnish

 This in vitro study was designed to prepare and evaluate a CPP-ACPF–containing varnish using high-purity reagents and precision laboratory instruments. Polyvinyl acetate and hydrogenated rosin gum were accurately weighed with an analytical balance (Sartorius, Germany). Each compound was separately dissolved in pure ethanol within glass beakers (Supertek, China) and stirred on a magnetic stirrer combined with a heater (Fater, Iran) at room temperature for 24 h. To prevent solvent evaporation, beaker openings were sealed with Parafilm M^®^.

 Afterward, the hydrogenated rosin gum solution, due to its lower viscosity, was gradually added to the polyvinyl acetate solution and mixed with a laboratory stirrer until a homogeneous consistency was obtained. Subsequently, fumed silica, sodium fluoride, xylitol, and casein phosphopeptide–amorphous calcium phosphate (CPP-ACP) were incorporated into the mixture. Before addition, the CPP-ACP powder was milled under a nitrogen atmosphere in a ball mill (Retsch, Germany) with 10 μm milling balls at 45 cycles/s for 30 min, yielding particles < 10 μm with a smooth texture. The final mixture was homogenized using a homogenizer (Mtops, South Korea), and a few drops of toffee flavor were added to improve palatability.

 All reagents were obtained from certified suppliers, and all procedures were conducted under standardized laboratory conditions to ensure reproducibility and accuracy.

###  Physicochemical Properties of the Varnish

####  Density Measurement

 In this study, after the preparation of the varnish formulation, a series of physicochemical and mechanical tests were conducted to evaluate the product’s properties. Initially, the density of the varnish was determined using a graduated cylinder and an analytical balance. For this purpose, the mass of the empty graduated cylinder was first measured and recorded. Then, exactly one milliliter of the varnish was added, and the total mass was measured again. The difference between the two measurements was considered the mass of the sample, and the density (ρ) in kg/m³ was calculated using the following formula:


(1) $$\rho =\frac{m}{v}$$

In this equation, (m) is the mass of the sample (kilograms) and (v) is the volume of the sample (cubic meters).

####  Viscosity Measurement

 To measure viscosity, a Brookfield viscometer (RVD-II Pro, USA) with spindle No. 14 was used. The test was conducted at 25°C with a rotational speed of 100 rpm. This device measures the fluid’s resistance to flow, i.e., viscosity, by rotating the spindle in the sample and recording the torque required to maintain a constant speed. This method allows for the measurement of a wide range of viscosities.

###  In Vitro Study on Enamel Samples

####  Sample Preparation

 To assess the effect of the varnish on the surface hardness of enamel, Vickers microhardness tests were performed on 36 sound premolar teeth that had been extracted within the past six months for orthodontic treatment. The teeth were initially examined under a 10 × optical microscope for cracks, hypoplasia, or white spot lesions, and defective samples were excluded. Then, for disinfection, the teeth were immersed in a 13% sodium Hypochlorite solution for 24 hours.

 The samples were mounted using self-curing acrylic resin (Poly methyl methacrylate) and Methyl methacrylate as the monomer solution in a silicone mold in such a way that the buccal surface was positioned horizontally and close to the edge of the mold. The separation of the buccal surface was performed using an ISOMET precision saw (Mechatronic, Germany) with a water-cooling system, and secondary cuts were made using a mini-unit turbine of the dental materials laboratory. The buccal surfaces were then remounted on acrylic bases and polished sequentially using disc abrasives and then 800-, 1200-, and 2000-grit sandpapers (Matador, Germany) with distilled water as a lubricant, until a smooth and flat surface was obtained.

 After preparation, the samples were randomly divided into three groups:

 Group 1: Prepared CPP-ACPF varnish

 Group 2: MI GC varnish

 Group 3 (Control): Varnish without active ingredient.

 Subsequently, the initial microhardness of the samples was measured using a Vickers microhardness tester (Bareiss, Germany) before demineralization step.

###  Demineralization Procedure

 For demineralization induction, a solution containing 2.16 mM CaCl₂, 2.2 mM NaH₂PO₄, and 50 mM acetic acid at pH 4.2 was prepared. Pilot tests indicated that the samples underwent demineralization within 3 hours in this solution. After this process, hardness was measured again.

 During the treatment stage, the corresponding varnishes were applied to the dried buccal surfaces using a fine brush, and the samples were immersed in artificial saliva for 24 hours. Then, the varnishes were removed using a surgical blade and swabs soaked in 50% acetone.

###  Treatment 

 All the specimens were stored for 10 days in a remineralizing solution containing 0.15-M KCl, 0.9-mM NaH₂PO₄, and 1.48-mM CaCl₂ at pH 7 (buffered with Tris). After this period, the final microhardness was recorded.

###  Scanning Electron Microscopy (SEM)

 To investigate surface morphology and structural changes, SEM imaging was performed at three stages (initial, after demineralization, and after remineralization) on selected samples from groups 1 and 2. Before imaging, the samples were coated with gold under vacuum using a sputter coater (Jeol, Japan) with argon gas. Images were captured with a S360 Cambridge SEM at 1000 × magnification, with careful adjustment of contrast, brightness, and resolution.

###  Microhardness Analysis

 Microhardness measurements of the tooth surface were taken at three stages: (1) baseline (sound enamel) before demineralization, (2) immediately after demineralization, and (3) after the remineralization period. The initial microhardness test was performed with a load of 200 g for 8 seconds. A square-based pyramidal diamond indenter with a 136° angle was pressed into the sample surface, and the two diagonal lengths of the indentation were measured using a light microscope. The hardness value was calculated using the following formula^23^:


(2) $$HV=\frac{F\times 1.854}{d^{2}}$$

In this formula, F is the applied load (in kilograms), d is the mean diagonal of the indentation (millimeters), and HV is the Vickers hardness number. One inherent limitation of the Vickers microhardness methodology is the creation of permanent indentations during baseline measurement. However, as all groups received identical indentation patterns and the study design is comparative rather than absolute, this limitation does not compromise the validity of the comparisons between groups.

###  Statistical Model: Repeated-measures Mixed ANOVA and Post Hoc Tests

 In this study, data from the Vickers microhardness test were analyzed to evaluate the effects of three types of dental varnishes at three time points. Given the study design, which involved repeated measurements on the same samples over time and comparisons between three independent groups, a repeated-measures mixed ANOVA model was employed. This statistical model can simultaneously examine intra-subject effects (changes over time), inter-subject effects (varnish type), and the interaction between these two factors on the dependent variable.

 The general form of the model is defined as follows^24^:


(3) $$Y_{ijk}=\mu +\alpha_{i}+\beta_{j}+{\left(\alpha \beta \right)}_{ij}+\varepsilon_{ijk}$$

*Y_ijk_*: The observed value of the dependent variable in group i, at time j, for subject k.

*µ*: overall mean of all observations

*α_i_*: inter-subject effect (e.g., Type of varnish)

*β_j_*: intra-subject effect (e.g., experimental stage: baseline, demineralization, remineralization),

*(αβ)_ij_*: interaction effect of time × group

*ε_ijk_*: random error, assumed to be normally distributed with mean zero and constant variance.

 Before running the model, its statistical assumptions were evaluated. The normality of the data distribution was assessed using the Shapiro–Wilk test:


(4) $$W=\frac{{\left(\sum_{i=1}^{n}a_{i}x_{i}\right)}^{2}}{\sum_{i=1}^{n}{\left(x_{i}-\bar{x}\right)}^{2}}$$

where *α_i_* is the fixed coefficient and *x_i_* is the ordered data. Homogeneity of variances between groups was assessed using Levene’s test.


(5) $$W=\frac{\left(N-K\right)}{\left(K-1\right)}.\frac{\sum_{i=1}^{k}N_{i}{\left(Z_{i0}-Z_{00}\right)}^{2}}{\sum_{i=1}^{k}\sum_{j=1}^{N_{i}}{\left(Z_{ij}-Z_{i0}\right)}^{2}}$$

where $Z_{ij}=\left|x_{ij}-\bar{x}\right|$ is the deviation from the median of group 2.

 Additionally, the sphericity assumption of the covariances was assessed using Mauchly’s test; if violated, the Greenhouse–Geisser or Huynh–Feldt correction was applied.

 After ensuring the assumptions or applying the necessary corrections, the mixed ANOVA model was performed. When the main or interaction effect was significant, post hoc tests were used to identify specific differences between groups and time points. The Tukey HSD test was employed for pairwise comparisons of means based on the following statistic:


(6) $$q=\frac{{\bar{X}}_{A}-{\bar{X}}_{B}}{\sqrt{\frac{MSE}{n}}}$$

In this equation, MSE denotes the mean squared error, and n is the number of samples per group. Additionally, the Bonferroni test was used to control Type I error rates in multiple comparisons.

 All statistical analyses were performed using SPSS 27, and a significance level of *P* < 0.05 was considered. The use of the mixed ANOVA model in this study, due to its ability to simultaneously analyze longitudinal changes and inter-group differences, significantly increased the accuracy and explanatory power of the results and enabled a scientific interpretation of the trend of changes in enamel microhardness under different intervention conditions.

## Results

###  Laboratory Findings

 The evaluation of the physicochemical properties of the varnishes showed that the density of the laboratory-produced varnish at 25°C was 1.2 g/mL, while the MI varnish exhibited a density of 1.1 g/mL. Viscosity measurements indicated that the laboratory-produced varnish, under experimental conditions (25°C, 100 rpm, 11.5% torque), had a viscosity of 1430‒1460 cP, demonstrating good stability and clinical applicability of the formulation. The same experiment was conducted under similar conditions for the MI varnish, which exhibited a viscosity range of 1410‒1450 cP. Based on the results of the t-test, this difference was not statistically significant (*P* = 0.06) (Table 1). Assessment of tooth surface hardness using the Vickers microhardness test revealed that the three groups (laboratory-produced varnish, MI varnish, and the control group) experienced a significant reduction in enamel hardness after demineralization. However, the degree of hardness recovery during the remineralization phase differed between the groups. In the first group treated with the laboratory-produced varnish, initial hardness values ranged from 447 to 552 VHN, which markedly decreased after demineralization to a minimum of 180.66 VHN. Following remineralization, enamel hardness increased significantly, reaching values between 296 and 403 VHN (Table 2). In the second group (MI varnish), initial hardness ranged from 421 to 507 VHN, with a severe decrease after demineralization to a minimum of 190.66 VHN. During remineralization, a moderate increase was observed, but final values remained lower than those of the first group (Table 3). In the control group, the mean initial hardness of samples ranged from 420 to 546 VHN, and demineralization caused a pronounced reduction to a minimum of 178 VHN. Remineralization resulted in only a slight increase, with final hardness values ranging from 278 to 327 VHN (Table 4).

**Table 1:** Viscosity test results

**Sample**	**Temperature (°C)**	**Speed (rpm)**	**Torque (%)**	**Viscosity cP**
MI Varnish (1)	25	100	11.5	1445
MI Varnish (2)	25	100	11.5	1413
MI Varnish (3)	25	100	11.5	1439
MI Varnish (4)	25	100	11.5	1444
MI Varnish (5)	25	100	11.5	1435
MI Varnish (6)	25	100	11.5	1431
MI Varnish (7)	25	100	11.5	1427
Prepared Varnish (1)	25	100	11.5	1448
Prepared Varnish (2)	25	100	11.5	1440
Prepared Varnish (3)	25	100	11.5	1433
Prepared Varnish (4)	25	100	11.5	1432
Prepared Varnish (5)	25	100	11.5	1456
Prepared Varnish (6)	25	100	11.5	1457
Prepared Varnish (7)	25	100	11.5	1452

**Table 2:** Microhardness test results after using the produced varnish

**Column**	**Initial hardness**	**DEM**	**REM**
1	474.0	244.3	303.7
2	492.3	231.7	377.3
3	472.3	226.3	296.0
4	475.3	222.0	313.3
5	473.3	180.7	281.7
6	525.3	237.0	376.3
7	525.3	218.0	347.7
8	493.7	268.0	375.0
9	476.0	200.0	398.0
10	476.3	213.0	351.0
11	436.7	190.3	348.0
12	449.7	222.3	347.3

**Table 3:** Microhardness test results after using the MI varnish

**Column**	**Initial hardness**	**DEM**	**REM**
1	471.0	233.3	241.0
2	443.7	245.7	246.0
3	468.3	185.3	312.7
4	447.7	223.0	277.0
5	509.7	246.7	352.7
6	457.3	190.3	315.7
7	468.7	248.0	312.0
8	489.3	233.7	335.7
9	471.3	152.3	260.7
10	490.0	204.7	344.0
11	500.7	160.3	255.3
12	499.3	210.3	385.3

**Table 4:** Microhardness test results after using the varnish without the active ingredient

**Column**	**Initial hardness**	**DEM**	**REM**
1	446.7	233.7	362.3
2	526.3	216.0	294.3
3	435.7	208.7	302.3
4	518.3	240.7	273.7
5	437.3	142.7	258.7
6	474.3	211.0	243.7
7	455.3	202.0	232.7
8	427.0	187.7	278.0
9	415.0	275.3	304.7
10	376.0	195.3	253.7
11	444.0	218.0	244.7
12	420.0	228.3	253.3

 SEM images (Figures 1, 2, and 3) also confirmed the microhardness findings. At baseline, all the samples exhibited a smooth, uniform surface. After demineralization, significant surface irregularities and roughness were observed, which corresponded with the decrease in surface hardness. During the remineralization stage, a relative improvement in surface uniformity was observed in both varnish-treated groups; however, the restoration quality was considerably higher in the group treated with the experimental varnish. SEM images of this group showed a more coherent, homogeneous reconstruction than the MI varnish, consistent with the microhardness test results. Evaluation of product stability over 12 months also indicated that the produced varnish maintained its physical characteristics, including color and consistency, under proper storage conditions (dark packaging and no light exposure).

**Figure 1:**
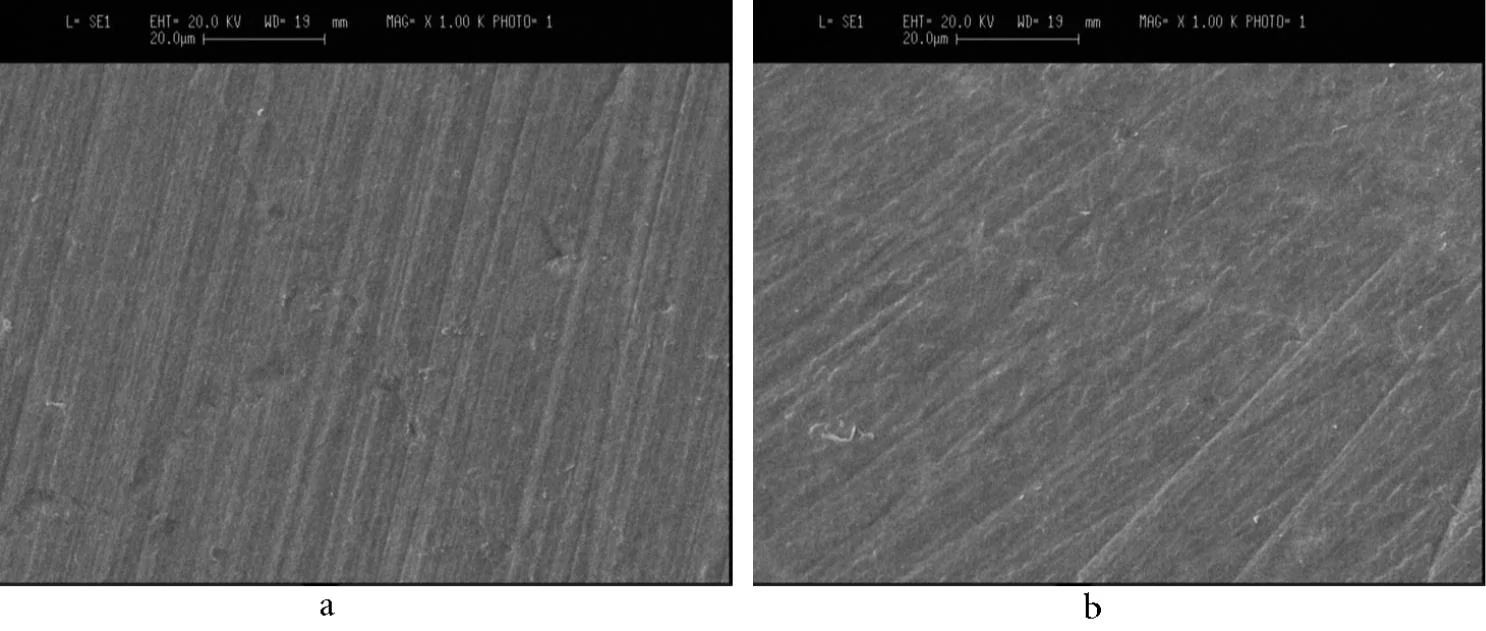
Scanning electron microscope (SEM) images at 1000 × magnification showing the enamel surface morphology at the baseline stage. (a) Sample from the CPP-ACPF varnish group. (b) Sample from the MI varnish group

**Figure 2:**
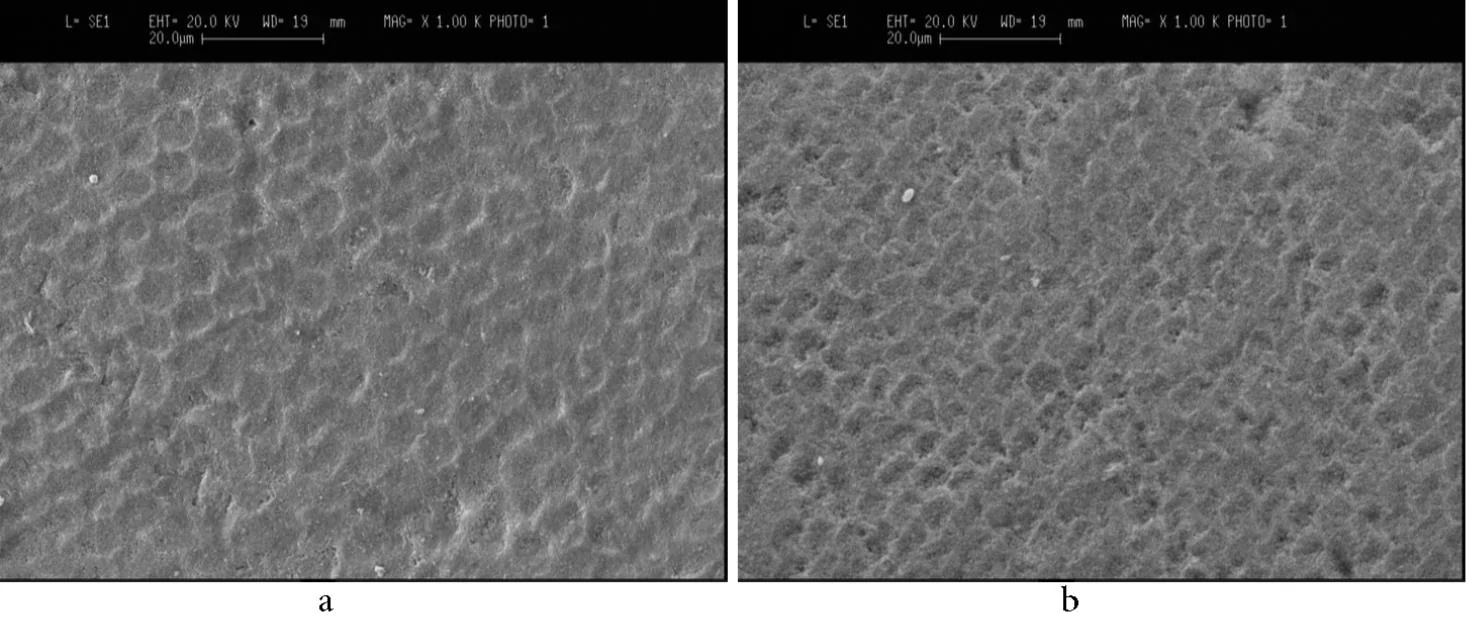
Scanning electron microscope (SEM) images at 1000 × magnification showing the enamel surface morphology after demineralization. (a) Sample from the CPP-ACPF varnish group. (b) Sample from the MI varnish group

**Figure 3:**
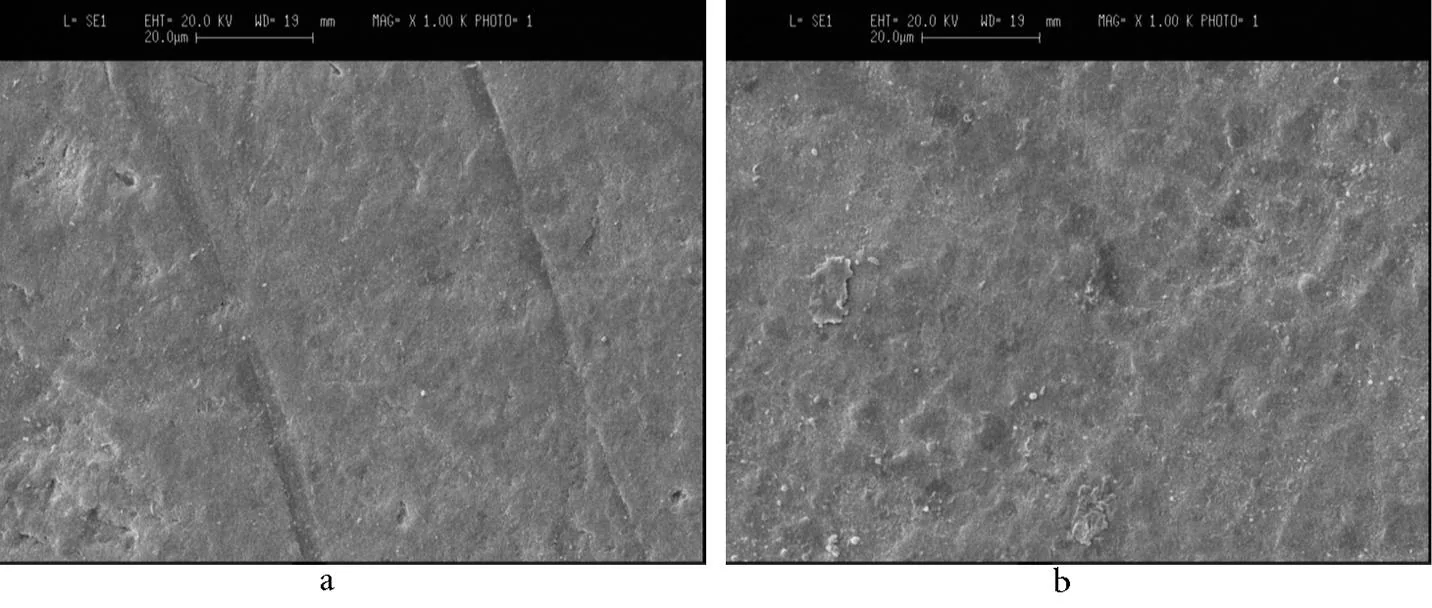
Scanning electron microscope (SEM) images at 1000 × magnification showing the enamel surface morphology after remineralization. (a) Sample from the CPP-ACPF varnish group. (b) Sample from the MI varnish group

###  Statistical Results

 The statistical indices of enamel microhardness at different experimental stages are presented in Tables 5 to 9 and Figures 4 and 5. At baseline, the group treated with the produced CPP-ACPF varnish exhibited the highest mean enamel surface hardness (480.11 ± 26.26 VHN), followed by the MI varnish group, which had a mean hardness of 476.42 ± 21.35 VHN. The control group showed a mean value of (448.00 ± 42.22 VHN). The relatively low standard deviation at this stage indicated a relatively uniform distribution of the data in the initial condition of the samples. After inducing demineralization, a considerable decrease was observed in all the groups, with mean hardness values decreasing to 221.14 ± 23.81, 211.14 ± 33.21, and 213.28 ± 32.18 for the produced varnish, MI varnish, and control groups, respectively.

**Table 5:** Results of assumption tests for the repeated-measures mixed ANOVA model

**Test type**	**Statistics**	**Value**	**Degrees of freedom**	**Significance level **	**Result**
Mauchly’s W	W	0.987	2	0.813	Assumption met
Equality of covariance matrices (Box’s M)	M	13.843	12, 5277.462	0.446	Assumption met
Homogeneity of variance (Levene) - initial stage	F	1.451	2, 33	0.249	Assumption met
Homogeneity of variance (Levene) - after demineralization	F	0.861	2, 33	0.432	Assumption met

**Table 6:** Mean and standard error of enamel hardness at three time points

**Time step**	**Average hardness (VHN) **	**Standard error**	**Lower 95% confidence interval**	**Upper 95% confidence interval**
Initial	468.176	5.207	457.970	478.382
DEM	215.185	5.005	205.375	224.995
REM	307.093	6.714	293.934	320.252

**Table 7:** Detailed results of the time*group interaction in the repeated-measures mixed ANOVA model

**Source of variance**	**Degrees of freedom (df)**	**F-statistic**	**Significance level**	**Effect size (Partial η^2^)**	**Interpretation**
Time*group (total)	4, 66	3.479	0.012	0.174	The pattern of change in stiffness over time differs between groups.
Linear contrast time*group	2, 33	2.617	0.088	0.137	The linear slope of recovery is not significantly different between groups.
Quadratic contrast time*group	2, 33	4.488	0.019	0.214	The acceleration of stiffness recovery is significantly different between groups.

**Table 8:** Inter-group effect on enamel microhardness

**Statistical index**	**Group 1 (produced CPP-ACPF varnish)**	**Group 2 (MI varnish)**	**Group 3 (control)**
VHN adjusted mean	348.065	330.241	312.148
Standard error	6.824	6.824	6.824
Lower 95% confidence interval	334.181	316.357	298.265
Upper 95% confidence interval	361.948	344.124	326.032

**Table 9:** Tukey HSD post hoc test results for pairwise comparisons between groups

**Pairwise comparison**	**Mean difference (I–J)**	**Standard error**	**Significance level**	**Lower 95% CI**	**Upper 95% CI**
MI, Produced CPP-ACPF	17.824	9.651	0.170	-5.860	41.504
Control, produced CPP-ACPF	35.917	9.651	0.002	12.236	59.597
Control, MI	18.093	9.651	0.162	-5.580	41.773

**Figure 4:**
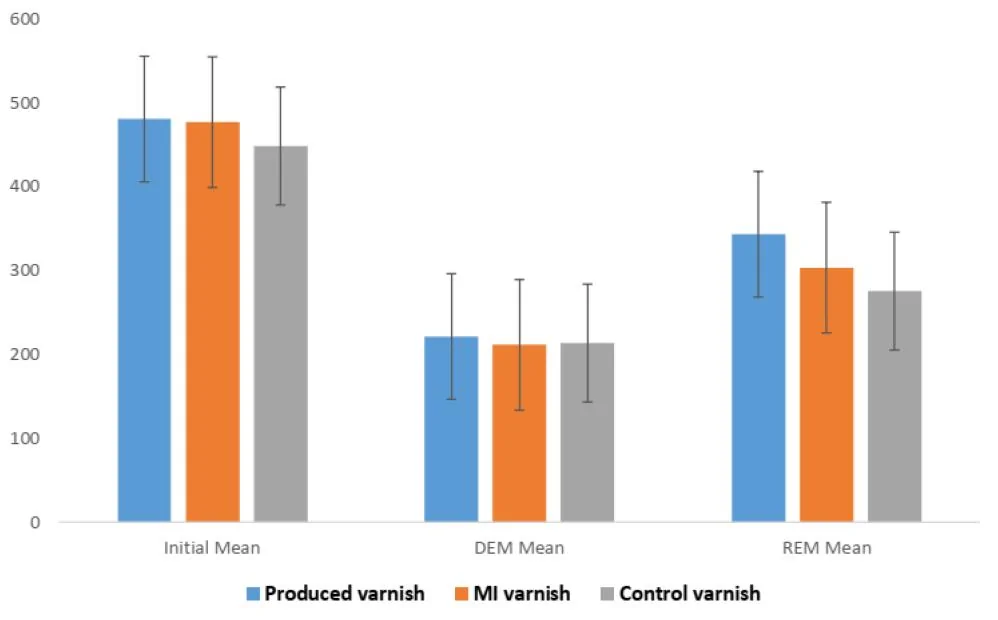
Mean enamel surface microhardness at baseline, post-demineralization, and post-remineralization across experimental groups

**Figure 5:**
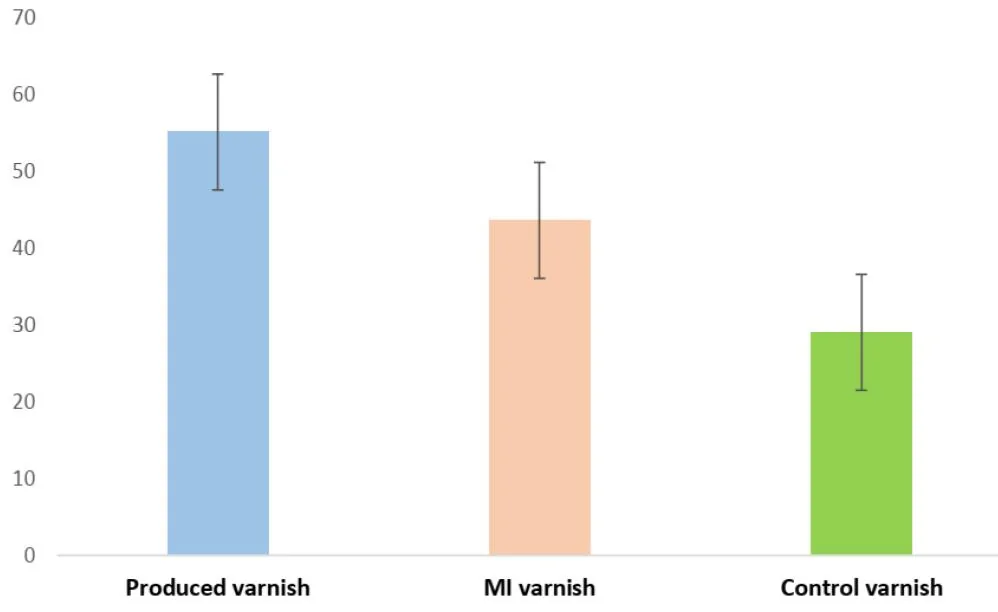
Percentage recovery of enamel surface microhardness among groups treated with CPP-ACPF varnish, MI varnish, and control

 During the remineralization stage, significant differences in hardness recovery were observed between groups. The highest mean was recorded in the produced varnish group (342.94 ± 36.76), followed by the MI group (303.17 ± 46.87) and the control group (275.17 ± 36.34). This difference highlights the CPP-ACPF formulation’s greater efficacy than that of the control group in restoring enamel surface hardness. Additionally, the lower standard deviation in the produced CPP-ACPF group compared to the other groups indicated greater stability and more consistent effectiveness of this formulation.

Figure 4 provides a visual comparison of mean enamel hardness across the three stages: baseline, demineralization, and remineralization. As shown, all three groups exhibited a sharp decrease during demineralization; however, during remineralization, the produced CPP-ACPF varnish group performed significantly better than the other two groups. The MI group showed moderate recovery, whereas the control group exhibited the least improvement and was unable to restore the initial hardness.

Figure 5 shows the comparison of enamel surface hardness recovery percentages between the groups. As illustrated, the group receiving the CPP-ACPF varnish showed the highest recovery, restoring approximately 55% of the lost hardness. This value was significantly higher than that of the MI varnish (43.59%) and control (29.02%) groups.

 Before conducting the repeated-measures mixed ANOVA model, the relevant statistical assumptions were examined to ensure the validity of the results (Table 5). The results of Mauchly’s test of sphericity indicated that the assumption of sphericity was met (W = 0.987, *P* = 0.813); therefore, no adjustment of the degrees of freedom using Greenhouse-Geisser or Huynh-Feldt corrections was required. Box’s test for the equality of covariance matrices across groups was also non-significant (M = 13.843, df = 12, 5277.462, *P* = 0.446), confirming that this assumption was satisfied. In addition, Levene’s test for homogeneity of error variances at each experimental stage showed no significant differences between groups; at the baseline stage (F = 1.451, *P* = 0.249) and after demineralization (F = 0.861, *P* = 0.432), the homogeneity assumption was upheld.

 After confirming the fulfillment of the statistical assumptions, the first intra-subject factor examined in the repeated-measures mixed ANOVA model was “time,” with the results presented in Table 6. This factor included three phases: baseline, demineralization, and remineralization, which simulated the healthy condition, the acidic environment, and the therapeutic or artificial saliva intervention, respectively. As expected, changes in enamel surface hardness across these three stages demonstrated a clear decreasing–increasing pattern.

 The interaction between time and group was also examined to determine whether the pattern of hardness changes across the three experimental phases was consistent across groups. According to the results presented in Table 7, the trajectory of hardness variation significantly differed between the three groups; specifically, the CPP-ACPF varnish group showed the steepest slope of hardness recovery, whereas the control group showed the slowest improvement, with the MI group occupying an intermediate position.

 The inter-group analysis also revealed that the mean enamel microhardness differed significantly between the three treatment groups (Table 8). The group receiving the produced CPP-ACPF varnish maintained the highest mean hardness (348.06 VHN) throughout the study period, while the control group showed the lowest value (312.15 VHN). The MI varnish group occupied an intermediate position (330.24 VHN), although its difference from the produced CPP-ACPF group was not statistically significant. These findings indicate that the type of intervention, independent of time, exerts a sustained effect on final enamel hardness. The CPP-ACPF formulation, by providing a stable source of calcium phosphate and fluoride ions, demonstrated greater efficacy than saliva-mediated natural remineralization.

 To accurately identify differences between groups, post hoc Tukey tests were performed (Table 9). The results showed a significant difference between the CPP-ACPF and control groups (*P* = 0.002), with the CPP-ACPF group maintaining a substantially higher mean hardness. The difference between the MI varnish and control groups was not significant (*P* = 0.162).

 Additionally, the difference between the produced CPP-ACPF and MI varnish groups did not reach statistical significance (*P* = 0.170).

 Overall, these findings indicate that the CPP-ACPF formulation demonstrated a clearly superior effect compared to natural remineralization, whereas MI showed intermediate performance.

## Discussion

 The present study demonstrated that the density of the prepared CPP-ACPF varnish was slightly higher than that of commercially available MI varnish. This difference is likely attributable to variations in the solid content ratio and polymer composition, reflecting the stability and uniformity of the newly developed formulation. Our findings are consistent with those reported by Bayrak et al.^18^ and Sleibi et al.^19^, both of which emphasized the importance of physicochemical properties, such as density and layer stability, in enhancing ion release and improving the clinical efficacy of varnishes. Therefore, it can be concluded that improving physical and chemical characteristics, including density, directly contributes to the therapeutic effectiveness of CPP-ACPF varnish.

 Furthermore, the present study showed that the viscosity of the prepared CPP-ACPF varnish ranged from 1430 to 1460 cP, providing an optimal balance between spreadability and adhesion to the tooth surface. This property allows the varnish layer to distribute evenly while maintaining resistance against rapid washout.

 The findings are consistent with those of Steinhaus et al.^20^ and Loumprinis et al.,^21^ who also highlighted the influence of resin and filler composition on viscosity and the importance of controlling environmental conditions. Therefore, the new formulation, with its optimized material composition, achieved a stable, effective viscosity.

 The present study further demonstrated that “time” plays a decisive role in changes in enamel microhardness, with a marked decrease during demineralization, followed by partial recovery during remineralization. This pattern aligns with previous studies, including those by Shen et al.^22^ and Olgen et al.,^13^ all of which emphasized the initial hardness loss and subsequent partial recovery after intervention. The three-stage design of the present study successfully simulated this clinical trend in a controlled and precise manner.

 Moreover, the findings indicated that the intensity and rate of remineralization were not uniform across the groups. Notably, in the CPP-ACPF-treated group, recovery of enamel hardness during the final stage occurred more rapidly and to a greater extent. This finding aligns with reports by Patel et al.,^23^ which confirmed a divergence in hardness recovery between CPP-ACPF and fluoride over longer time intervals.

 Clinical studies have also emphasized the superiority of CPP-ACPF-containing formulations compared to pure fluoride, showing that this compound not only increases enamel hardness but also enhances its long-term resistance to acidic attacks. Microhardness and SEM analyses demonstrated that both varnishes promoted remineralization; however, the experimental CPP-ACPF varnish produced higher surface hardness and more uniform enamel compared to MI varnish. These findings are consistent with studies by Majithia et al.^24^ and Kooshki et al.,^25^ which confirmed the role of CPP-ACPF in improving enamel hardness. The superiority of the experimental varnish is likely attributable to its optimized chemical composition and structural stability, making it a suitable alternative to imported products.

 Overall, the experimental varnish can be considered an effective, stable, and cost-efficient option for caries prevention programs, particularly for high-risk populations. To further substantiate the findings and enhance the performance of the produced varnish, several recommendations for future research are proposed. Firstly, while the varnish has demonstrated efficacy in vitro, its clinical performance in real oral conditions warrants investigation. This should include evaluation in high-risk groups, such as children and patients with xerostomia, to gain more accurate data regarding its clinical effectiveness, retention time, and patient acceptance. In addition, it is crucial to assess the biocompatibility and tissue safety of the varnish. Specifically, studies focusing on the potential cytotoxicity and inflammatory effects on gingival cells and surrounding soft tissues will help establish a comprehensive safety profile for clinical application.

 Furthermore, it would be beneficial to compare the experimental varnish with more advanced formulations that incorporate nanoparticles or bioactive compounds, such as bioglass, nano-hydroxyapatite, or chitosan derivatives. These comparisons could offer valuable insights into how these novel materials might improve the varnish’s efficacy.

 Another important avenue for future research is the evaluation of the varnish’s physical and chemical stability under various environmental conditions, such as high temperature and humidity. Simulating real-world storage and transportation conditions is essential for determining its shelf life and ensuring its feasibility for large-scale distribution.

 Finally, it is recommended to investigate the effect of different application frequencies and intervals on enamel remineralization. Understanding the optimal number and timing of applications, whether single or repeated, will help establish the most effective clinical usage protocol for the varnish.

## Conclusion

 The findings of this study demonstrated that the experimentally produced CPP-ACPF varnish exhibited physical properties and remineralization performance comparable to commercial counterparts. This varnish significantly increased enamel surface hardness after demineralization and provided more uniform and efficient remineralization than MI varnish, as confirmed by microhardness testing and SEM imaging. Additionally, a 12-month stability assessment showed consistent color, texture, and homogeneity of the product. These results, in line with previous studies, highlight the critical importance of precise formulation and appropriate design in enhancing the efficacy of dental varnishes.

## Competing Interests

 The authors declare that they have no competing interests.

## Ethical Approval

 This article is based on a part of a PharmD thesis with ethical code of IR.ABZUMS.REC.1403.052, Faculty of Pharmacy, Alborz University of Medical Sciences, Karaj, Iran.
